# An In-Line Photonic Biosensor for Monitoring of Glucose Concentrations

**DOI:** 10.3390/s140915749

**Published:** 2014-08-02

**Authors:** Ala'aldeen Al-Halhouli, Stefanie Demming, Laila Alahmad, Andreu LIobera, Stephanus Büttgenbach

**Affiliations:** 1 Institute of Microtechnology, Technische Universität Braunschweig, Langer Kamp 8, 38106 Braunschweig, Germany; E-Mails: stefanie.demming@googlemail.com (S.D.); lailadtr@yahoo.com (L.A.); s.buettgenbach@tu-bs.de (S.B.); 2 Mechatronics Engineering Department, German Jordanian University, 11180 Amman, Jordan; 3 CentreNacional de Microelectrònica (IMB-CNM, CSIC), 08193 Barcelona, Spain; E-Mail: andreu.llobera@imb-cnm.csic.es

**Keywords:** glucose, enzymes, immobilization, beads, biosensors

## Abstract

This paper presents two PDMS photonic biosensor designs that can be used for continuous monitoring of glucose concentrations. The first design, the internally immobilized sensor, consists of a reactor chamber, micro-lenses and self-alignment structures for fiber optics positioning. This sensor design allows optical detection of glucose concentrations under continuous glucose flow conditions of 33 μL/h based on internal co-immobilization of glucose oxidase (GOX) and horseradish peroxidase (HRP) on the internal PDMS surface of the reactor chamber. For this design, two co-immobilization methods, the simple adsorption and the covalent binding (PEG) methods were tested. Experiments showed successful results when using the covalent binding (PEG) method, where glucose concentrations up to 5 mM with a coefficient of determination (R^2^) of 0.99 and a limit of detection of 0.26 mM are detectable. The second design is a modified version of the internally immobilized sensor, where a microbead chamber and a beads filling channel are integrated into the sensor. This modification enabled external co-immobilization of enzymes covalently onto functionalized silica microbeads and allows binding a huge amount of HRP and GOX enzymes on the microbeads surfaces which increases the interaction area between immobilized enzymes and the analyte. This has a positive effect on the amount and rate of chemical reactions taking place inside the chamber. The sensor was tested under continuous glucose flow conditions and was found to be able to detect glucose concentrations up to 10 mM with R^2^ of 0.98 and a limit of detection of 0.7 mM. Such results are very promising for the application in photonic LOC systems used for online analysis.

## 1. Introduction

The rapid development in microsystems technology, and its suitability for realizing microfluidics devices, has led to the opening of novel aspects in biomedical and life science applications. Accordingly, miniaturization towards lab-on-a-chip has devoted great interest on many researchers. As a result, many miniaturized devices including: pumps, dispensers, biosensors have been presented recently [1,2].

Among these devices, micro biosensors have significant and important impact on the development of lab-on-a-chip systems since they are optimal candidates for detecting cells, antibodies, DNA, and different substrates such as glucose, ethanol and hydrogen peroxide [3].

The basic principle of biosensor depends on the interaction of analyte in sample with the biological element (it can be whole cell or part of it, such as enzymes, antibodies or nucleic acids). This biological element is attached basically to an interface like the membrane, at which a specific reaction between the biological element and the analyte takes place. As a consequence of this reaction, a specific signal is developed and transformed through the transducer into another signal, which can be more easily measured and analysed through the detector.

Most regularly used elements are enzymes that may be used in purified form, immobilized form or may be present in microorganisms. In this case sensors are named as enzyme biosensors. Enzyme biosensors can be classified according to the type of substrate that reacts with the enzyme to yield a product. Determination of glucose, H_2_O_2_ and ethanol are examples of such sensors.

The idea of a glucose enzyme electrode was first proposed in 1962 by Clark and Lyons [4]. Since that time, excellent economic prospects and fascinating potential for basic research have led to many sensor designs and detection principles for the biosensing of glucose [5].

The evolution of glucose biosensors was started by introducing the first glucose oxidase (GOX) biosensor. GOX has been immobilized in polyacrylamide gel on a gas permeable membrane covering the electrode [6]. In this work, the decrease in O_2_ concentration using a Clark oxygen electrode was electrochemically measured. This decrease was used as indicator for the glucose concentration.

Due to the detected vibration in oxygen level, this method was replaced by measuring electrochemically the H_2_O_2_ oxidation products [7]. But detecting H_2_O_2_ or oxygen at high positive potentials causes oxidation of interfering molecules, which made this type of biosensors not totally adequate for measuring glucose concentration.

To solve this challenge, a better solution was presented in a second generation, in which the oxygen is replaced by other oxidising agents (mediators) [8]. These mediators have appropriate oxidation potentials and their concentrations could be controlled. They can react with the active site of the enzyme and with the electrode surface, shuttling the electrons between the enzyme and the electrode. The electrochemical signal is detected depending on the oxidation of these mediators. This type of system makes the sensing independent of oxygen. In addition, they have the advantage that if the oxygen is deficient in a system, the glucose concentration can be measured independently from oxygen.

An optical microchip biosensor was used to determine the dynamic release of glucose and ethanol produced from sucrose by immobilised yeast cells [9]. The co-immobilization of either glucose oxidase–horseradish peroxidase (GOX-HRP), or alcohol oxidase–horseradish peroxidase (AOX-HRP) was performed to conduct the experiments. Results showed that the two cell products (glucose and ethanol) could be continuously and quantitatively monitored by flow-through this sensor with the co-immobilised enzymes, which catalysed a system of reactions ending with the HRP catalysed chemiluminescence oxidation of luminol, enhanced by *p*-iodophenol.

It has also been found that the GOX-HRP biosensor could be used during five days without any significant loss of activity, whereas the AOX-HRP sensor needed to be continuously corrected for loss of activity with time.

On the other hand, an integrated optical glucose sensor fabricated using PDMS waveguides on a PDMS substrate was successfully produced and tested [10]. The waveguides are thermally-defined monolithic PDMS waveguide systems, fabricated on a PDMS substrate. The surface of such waveguides was then treated. Afterwards, they were connected to an LED source that injected light into the waveguides at a 450 nm peak wavelength. The oxygen-sensitive ruthenium dye absorbs strongly at this wavelength, and emits light at a peak wavelength of 610 nm. The sensing system used the combination of oxygen sensitive dye tris(2,2′-bipyridyl) dichlororuthenium(II) hexahydrate and glucose oxidase. The dye/enzyme system was immobilized onto the surface of the waveguides using layer-by-layer self-assembly. Changes in the enzyme/dye system as it interacts with the surrounding environment are monitored using end-face interaction with light injected into waveguides. Results with too low overall sensitivity were reported by the sensor. This low sensitivity of the sensor was related to the low fluorescence intensity caused by both the failure of the vane demolding and a general lack of sensitivity of the spectrometer.

Recently, investigations towards miniaturizing biosensors into mini/micro-scale devices are a trend topic. This provides the advantages of fast analysis, parallelization, low cost, portability and minute reagent consumption.

This work presents the implementation of a photonic glucose biosensor based on internal and external co-immobilization of glucose oxidase (GOX) and horseradish peroxidase (HRP). The internal co-immobilization was performed on the internal PDMS surface of the sensor chamber while the external co-immobilization was on functionalized silica micro-beads. The external immobilization of microbeads has several advantages such as the increase of the enzymatic activity due to their high surface area to volume ratio. In addition, since large number of beads can be immobilized and used for several chips, reductions in reagent costs and experimentation time are obvious in comparison with direct immobilization techniques [11] for each tested PDMS chip.

## 2. Theory

Enzymes are proteins which act in a highly specific biocatalytic way and can therefore accelerate biochemical reactions through decreasing its activation energy. One advantage of using enzymes is that they can survive several reaction cycles without changing their structural configuration. For the detection of glucose the most employed enzymatic components are glucose oxidase or glucose dehydrogenase.

The oxidase enzymes work through catalysing a reaction where the substrate is oxidized by molecular oxygen producing hydrogen peroxide, which can be easily detected by various detection methods [4]. Glucose oxidase catalyses the oxidation of glucose to gluconic acid and H_2_O_2_ in the presence of oxygen (Equation (1)). Accordingly, the glucose concentration can be predicted indirectly by measuring either the depletion of O_2_ or the resulting H_2_O_2_.

(1) $$\text{Glucose}+{\text{O}}_{2}\overset{\text{GOX}}{\to }\text{gluconic acid}+{\text{H}}_{2}{\text{O}}_{2}$$

In this study, the H_2_O_2_ is considered as an indicator for determination of the glucose concentration. H_2_O_2_ is correlated with HRP which is a kind of peroxidase, a popular enzyme that has been widely used in biochemical applications [4]. HRP usually catalyses the reaction developed between H_2_O_2_ and added substrate that is chromogenic or luminescence. The substrate is oxidized to its oxidized form and H_2_O_2_ is reduced into H_2_O according to the following equation:

(2) $${\text{H}}_{2}{\text{O}}_{2}+\text{substrate}\overset{\text{HRP}}{\to }{\text{H}}_{2}\text{O}+\text{oxidized substrate}$$

3,3′,5,5′-Tetramethylbenzidine (TMB) is considered as a substrate in all experiments. Through detecting the oxidized form of the TMB, the analyte concentration is optically determined.

## 3. Materials and Methods

### 3.1. Materials

The photoresists SU-8 5 and SU-8 50 and their development solution propylene glycol methyl ether acetate (PGMEA) were purchased from MicroChemCorporation (Newton, MA, USA). The PDMS Sylgard 184 elastomer kit was bought from Dow Corning (Midland, MI, USA) and used according to the datasheet. The GOX (product G7141-50KU), 3,3′,5,5′-tetramethyl­benzidine (TMB), acetate buffer solution (pH 4.6), HRP type VI, sodium acetate buffer, phosphate buffered saline (PBS) (P3813) and glutaraldehyde were purchased from Sigma-Aldrich Co. (St Louis, MO, USA).

### 3.2. Fabrication

The photonic glucose biosensor is fabricated by applying UV-depth lithography for the fabrication of the SU-8 photoresist negative master and soft lithography processing for PDMS molding. The master is prepared by patterning double photolithographic processes onto a 700 μm thick soda-lime glass substrate. The fabrication process begins by cleaning and dehydrating the substrate on a hotplate for 1 h at 120 °C followed by oxygen plasma activation in a barrel etcher.

The first photolithographic process starts with spin-on a thin layer of SU-8 5 on the substrate at 3000 rpm for 30 s and simultaneously drying it for 10 min at 95 °C. After that, SU-8 layer is flood exposed to UV-light and baked at 95 °C for 10 min. This layer is about 5 μm in thickness and acts as an adhesion promoter and seed layer for the next structure. Before spin coating of the structure layer, the seed layer is activated in oxygen plasma.

The sensor body is then patterned by processing SU-8 50 photoresist two times. In each step 4 mL of the photoresist is spun at 1200 rpm, levelled at planar plate and then dried at 95 °C. A total layer thickness of 230 μm is obtained after the simultaneous processing of the SU-8 50. After that, the substrate is exposed to UV-light for 100 s and baked for 20 min at 95 °C. Exposed master is then left over night and developed the next day in propylene glycol methyl ether acetate (PGMEA, MicroChem, Newton, MA, USA).

Replica of this master is formed by using PDMS (Sylgard 184 elastomer kit, Dow Corning, Midland, MI, USA). The PDMS pre-polymer is prepared by mixing the curing agent with elastomer base in a 1:10 ratio (volume:volume). The mixture is then degassed to remove air bubbles, poured onto the master, heated at 80 °C for 30 min and finally peeled off from the master. PDMS biosensor molds are then bonded to glass chips and connected with inlet and outlet ports.

### 3.3. Co-Immobilization Methods

The success of enzymes (HRP, GOX) co-immobilization is one of the critical factors that influence the glucose sensor performance. Several co-immobilization methods, simple adsorption, covalent binding (PEG) on the internal PDMS chamber surface and enzymes external covalent binding onto functionalized silica microbeads have been examined. Co-immobilization processes are described in the following subsections.

#### 3.3.1. Internal Co-Immobilization on PDMS

This method aims at direct covalent immobilization of HRP and GOX on the internal PDMS surface of the sensor chamber. To examine the efficiency of enzymes immobilization on PDMS surface, two procedures were tested: the simple adsorption, and the polyethylene glycol (PEG)/11-triethoxysilylundecanal (11-TESU).

The simple adsorption method was the simplest and the fastest. In this process, 0.001 mg HRP and 0.001 mg GOX were dissolved in PBS and then simply incubated in the sensor chamber at room temperature for 1 h.

The implementation of the other procedure using PEG aimed at surface treatment to avoid non-specific binding of the enzymes to PDMS and to improve the immobilization performance.

PEG is a linear polymer consisting of repeated units of –CH_2_–CH_2_–O. The polymer is well soluble in a number of organic polar and apolar solvents, as well as in water. Owing to its simple structure and chemical stability, it is a prototype of an inert, biocompatible polymer. PEG is used for non-ionic surfactants and as an additive in cosmetics, pharmaceuticals and food. When bound to surfaces [12], PEG repels other molecules by steric effects; the incoming molecule is not attracted by e.g., electrostatic force and cannot penetrate the hydrated PEG layer. This results in inert hydrophilic surfaces with less ‘stickiness’ [13]. The PEG act as detergents to prevent the non-specific binding of the enzyme (HRP) to the surface.

The co-immobilization procedure begins by careful washing of the internal sensor chamber with ethanol and distilled water. After that, 1 mg of PEG/PVA is dissolved in a 1 mL of distilled water. PEG dissolved simply in water, but PVA is dissolved in a hot water. Dissolved solution is then incubated in the washed chamber at room temperature for 1 h.

To rinse PEG/PVA, a salinization process using 2% triethylamine (TEA) and 2% 11-TESU solutions was performed. The procedure begins by dissolving a 1 mL of TEA in 45 mL distilled water and a 1.1 mL TESU in 48.9 mL ethanol. The two solutions are then well mixed (1:1) together and incubated in the system for 1 h at room temperature. After that the system is washed with ethanol and heated at 80 °C onto a hotplate for about 2 h. During this time, 1 mg HRP, 1 mg GOX and 1 mg sodium cyanoborohydride are dissolved in 10 mL PBS. Finally, prepared solution is incubated in the system for 1 h.

#### 3.3.2. External Co-Immobilisation on Microbeads

The external co-immobilization method aims at covalent immobilization of HRP and GOX on the surface of functionalized silica microbeads. The enzymes HRP and GOX are first immobilized and then the beads are inserted to the beads filling chamber.

The immobilization procedure begins by mixing 10 mL of 2.5% of glutaraldehyde solution with 1.5 g of silica beads under shaking at room temperature (RT) for 90 min. After that, mixed solution was filled up with DI-H_2_O and mixed again. Filled tubes were then centrifuged for 10 min at full speed. The over suspended layer was discarded. This centrifugation procedure is repeated 3 times. In parallel, the enzyme solution was prepared by dissolving 10 units of HRP and 10 units of GOX in 0.1M PBS. The external co-immobilization was achieved through adding the enzymes solution to the previously prepared beads and maintaining them for 1 h at RT. The solution is then incubated at 4 °C until use.

The introduction of external co-immobilization on the surface of the microbeads presents attractive advantages among other internal immobilization methods. The high increase of the surface area to volume ratio enables the immobilization of a huge amount of enzymes at the microbeads surface and accordingly a large increase in the interaction area between immobilized enzymes and the analyte. This has a positive effect on the amount and rate of chemical reactions taking place inside the chamber. In addition, external immobilization offers strong covalently enzymes adhesion to the microbeads surface (due to the use of glutaraldehyde solution).

### 3.4. Sensor Design

The photonic biosensor was microfabricated using softlithography in PDMS according to the methods described in previous section. It comprises a glass substrate and a biosensor body.

**Figure 1.**
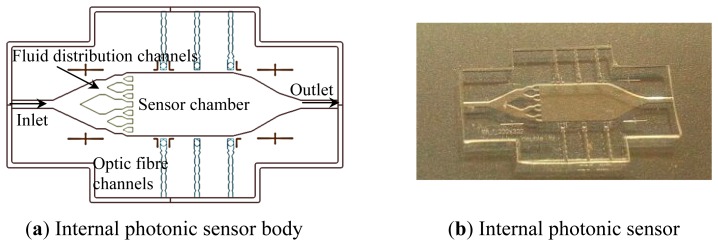
Internal photonic biosensor (**a**) schematic of the sensor body (**b**) photo of the internal photonic biosensor

The internal biosensor body consists of the sensor chamber fluid inlet and outlet and distribution and optic fibre channels (Figure 1b). The sensor body contains the inlet and outlet channels, which allow the fluid flow through the sensor chamber. The fluid distribution channels are arranged in a manner to achieve homogenous distribution of the fluid in the chamber. Optic fibre channels are located perpendicular to the sensor chamber. These channels are found in a buckle shape in order to enable fixing the optic fibres in the channel. These channels ended with biconcave lenses, by which light can be concentrated and then transmits through the solution toward the receptor fibre (Figure 1a).

**Figure 2.**
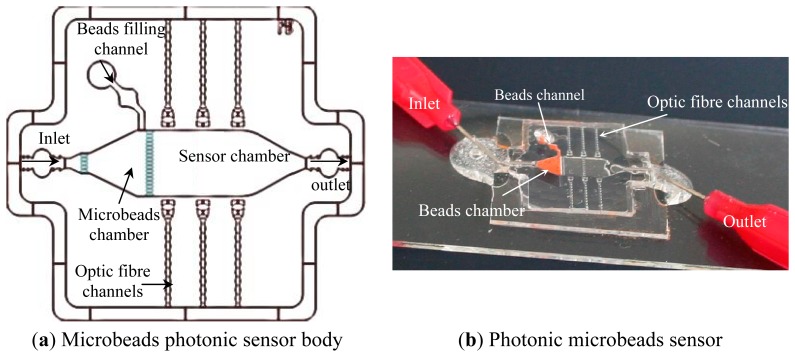
External microbeads photonic sensor (**a**) schematic of the sensor body (**b**) photo of the photonic biosensor including immobilized microbeads.

The external biosensor body consists of the sensor chamber, microbeads filling chamber, fluid inlet, outlet and optic fibre channels (Figure 2a). The microbeads chamber is located between the inlet channel and the sensor chamber and bordered by narrow grids. The immobilized beads are inserted through a separate beads filling channel that is connected to the beads chamber to enable inserting immobilized beads directly. Fibre optic alignment channels are located perpendicular to the sensor chamber and ended with biconcave microlenses. Their buckle shape enables smoother entering of the optic fibres into the channels, while the biconcave lenses correct the numerical aperture of the fiber optics. A photo of the photonic biosensor including immobilized microbeads is shown in Figure 2b.

## 4. Experiments and Results

### 4.1. Measurement Set-Up

Photonic biosensors were characterized experimentally by using two multimode optical fibres of 230 μm diameter (Thorlabs, Dachau, Germany), a monochromatic light source (S1FC635, Thorlabs), and a spectrometer (Oceanoptics HR4000, Dunedin, FL, USA). The two fibre optics are first inserted into the PDMS self-alignment fibre optic channels (Figure 2), where one of them is connected to the light source while the other one is connected to the spectrometer. Therefore, light-analyte interaction has an optical path equal to the width of the reactor. If the analyte is properly diluted in the media and has no photonic re-emission, then the performance of the proposed photonic biosensors follows the Beer-Lambert law, where the absorbance is proportional to the optical path, the concentration and the molar absorptivity.

### 4.2. Testing and Results

In this study, the internal and external immobilization sensor designs have been tested. For the internal immobilization design, the simple adsorption and PVA/PEG procedures were examined.

The simple adsorption method was tested after direct immobilization of enzymes on the PDMS surface and rinsing the system with PBS. Measurements of the optical absorbance using TMB mediator have been conducted, where the mediator changes its colour to green due to the reduction of H_2_O_2_ (Equation (2)). The proportional change of colour depends on the optical absorbance and provides information regarding the glucose concentration. Experimentally, several glucose concentrations were continuously pumped at a 33 μL/h and results were reported after fixed time intervals. Tests showed fluctuations in the optical absorbance measurements and no effective measurements were achieved. This has been related to the non-specific binding of the enzymes to the surface due to the lack of surface pre-treatment.

**Figure 3.**
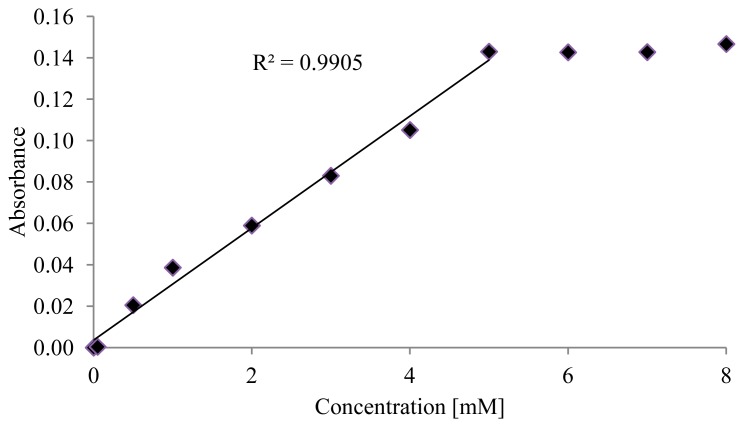
Glucose absorbance calibration curve after 15 min run.

Accordingly, the second method using PEG procedure followed by a salinization process has been examined. The same previous testing procedure has been conducted. As shown in Figure 3, absorbance results are plotted as a function of glucose concentration and linearly fitted. Results showed that the sensor is able to detect glucose concentrations up to 5 mM with R^2^ of 0.99 and a limit of detection of 0.26 mM.

**Figure 4.**
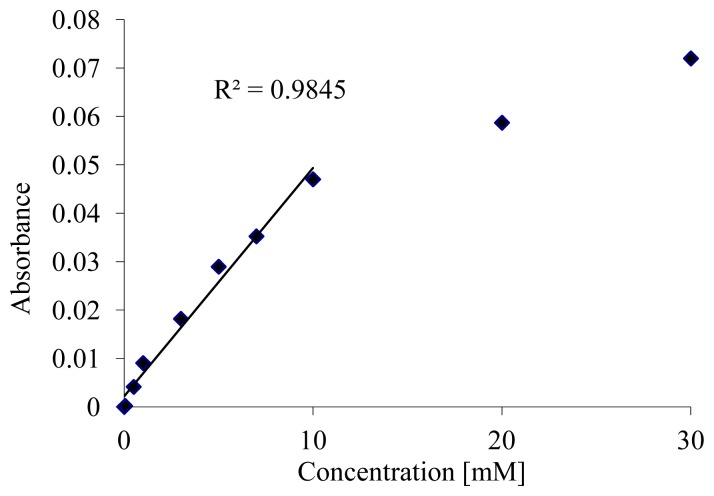
Glucose absorbance calibration curve after 10 min run.

These results proved that the surface modification and salinization steps were effective and improved the sensor performance. However, the amount of immobilized enzymes is limited and influences the biosensor detection range. For this purpose, a modified photonic biosensor design including beads chamber was tested. The beads chamber was firstly filled with the immobilized microbeads via the filling channel. Then, this channel was sealed and the detection protocol was started. The same measurement procedure was implemented here. As it is shown in Figure 4, absorbance results are plotted as a function of glucose concentration and linearly fitted. Results show that the sensor is able to detect glucose concentrations up to 10 mM with R2 of 0.98 and a limit of detection of 0.7 mM.

External immobilization sensor provided higher range of concentration measurements, which were obtained through averaging more than one spectrum, than that obtained by the internal design. This is related to the high increase in the immobilization surface area to the volume ratio which increases the interaction area between the enzymes immobilized on the beads and the analyte. So, it can be considered that the external immobilization design uses simpler and more efficient immobilization strategy. Moreover, the utilized external immobilization strategy allows simpler immobilization procedure for more than one type of enzymes at the surface of the beads, and thus different substrates can be detected. From obtained photonic results, it can be also concluded, that using glutardialdehyde as a crossing reagent for external immobilization of enzymes into beads increases the strength of binding of enzymes to beads, which adds a positive effect on their activity and specificity to substrate. This can be related to the maintaining of the conformations and the active sites of the enzymes or their very slight change during the immobilization step.

Finally, it is worth mention that besides the sensor design itself, the used immobilization strategies strongly influence the performance and the sensitivity of the tested biosensor. Summary of the sensor designs results are listed in Table 1.

## 5. Conclusions

This paper presented two photonic glucose biosensor designs based on internal and external co-immobilization procedures of glucose oxidase (GOX) and horseradish peroxidase (HRP) under continuous flow conditions. The implemented co-immobilization methods were explained. Results showed that the internal photonic biosensor design enables detection of glucose concentrations up to 5 mM with R^2^ of 0.99 and a limit of detection of 0.26 mM. However, the introduction of the external immobilization method offers the possibility of immobilizing huge amount of enzymes covalently to the microbeads which influenced positively the amount and rate of chemical reactions taking place inside the biosensor chamber and enables continuous flow detection of glucose concentrations up to 10 mM with R^2^ of 0.98 and a limit of detection of 0.7 mM. Such results are very promising for the application in photonic lab-on a chip systems used for online analysis, where the general challenge of such systems is the lack of reliable data on reactant and product concentrations across the downstream of the micro-bioreactors. This is due to the extremely small volumes, which are difficult to analyze using conventional offline analysis like HPLC.

## Figures and Tables

**Table 1.** Summary of the different sensor designs performance and co-immobilization methods.

**Sensor**	**Co-Immobilization Method**	**R^2^**	**LOD**	**Detection Range up to**
Internal Sensor	Simple adsorption	‐	‐	‐
Internal Sensor	PEG covalent binding	0.99	0.26	5 mM
External Sensor	PEG covalent binding + glutaraldehyde	0.9845	0.7	10 mM
